# An evidence-based evaluation on the use of platelet rich plasma in orthopedics – a review of the literature

**DOI:** 10.1051/sicotj/2017036

**Published:** 2017-10-09

**Authors:** Nasir Hussain, Herman Johal, Mohit Bhandari

**Affiliations:** 1 Central Michigan University College of Medicine, CMED Building 1280 S. East Campus St. Mt. Pleasant MI 48859 USA; 2 Division of Orthopaedic Surgery, Department of Surgery, McMaster University & Centre for Evidence Based Orthopaedics 293 Wellington Street North, Suite 110 Hamilton Ontario L8L 8E7 Canada

**Keywords:** Platelet rich plasma, Orthobiologics, Evidence-based medicine, Review

## Abstract

Within orthopedics, the use of platelet-rich plasma (PRP) has been rapidly increasing in popularity, however, its true effectiveness has yet to be fully established. Several studies find that injecting PRP to the site of injury does not provide any significant benefit with respect to clinical outcomes; however, many others report the contrary. Due to the conflicting evidence and multiple meta-analyses conducted on the topic, a literature review of high-quality evidence on the use of PRP for common orthopaedic conditions was performed. Thus far, the evidence appears to suggest that PRP may provide some benefit in patients who present with knee osteoarthritis or lateral epicondylitis. On the other hand, evidence appears to be inconsistent or shows a minimal benefit for PRP usage in rotator cuff repair, patellar and Achilles tendinopathies, hamstring injuries, anterior cruciate ligament (ACL) repair, and medial epicondylitis. There is limited confidence in the conclusions from the published meta-analyses due to issues with statistical pooling, and limited subgroup analyses exploring the substantial heterogeneity across studies. Evidence-based clinicians considering the use of PRP in their patients with musculoskeletal injuries should be weary that the literature appears to be inconsistent and thus far, inconclusive.

## Platelet-rich plasma in orthopedics

Within orthopedics, the use of platelet-rich plasma (PRP) has been increasing in popularity. United States estimates alone suggest that approximately 86,000 athletes are treated with PRP annually [1]. Even though its popularity is rising, its true effectiveness has yet to be fully established. Several studies find that injecting PRP to the site of injury does not add any significant benefit to clinical outcomes; however, many others report the contrary. This becomes even more of a concern since the cost of treatment can be relatively high. Peerbooms et al. (2010) reported that the cost for a single PRP injection is approximately $840.00 USD whereas a simple corticosteroid injection is around $300.00 USD [2]. With the conflicting evidence and high cost of PRP treatment, it is imperative that a more definitive answer regarding its efficacy is found. Given the continued uncertainty of PRP with regard to its efficacy at improving various clinical outcomes in a broad spectrum of orthopedic conditions, we undertook this review to help clinicians better understand the basics behind PRP and the clinical evidence surrounding it.

### What is platelet-rich plasma?

The platelets contained within autologous blood play an important role in healing since they secrete several growth factors to the site of injury [3]. Briefly, among other roles, these platelets serve to promote mitogenesis of healing capable cells and angiogenesis in the tissue [4]. Autologous blood, which contains such platelets in higher than normal concentrations, is commonly referred to as platelet-rich plasma (PRP). For instance, the normal platelet count in healthy individuals is around 1.5–4.5 × 10^5^/μL; however, to be considered PRP, the platelet should be 4–5 times above this amount [5]. This relatively recent biotechnology has been reported to enhance the healing process since an increased number of platelets results in an increased number of secreted growth factors, thereby theoretically improving the healing process [4, 6]. Some of the growth factors in PRP include: platelet-derived growth factor (PDGF), transforming growth factor beta (TGF-β), vascular endothelial growth factor (VEGF), and epithelial growth factor (EGF) [1, 3, 6]. Thus, unlike recombinant technology which is synthetic, PRP takes advantage of the naturally occurring proteins in the healing process. In addition to these factors, PRP contains adhesion molecules which promote bone formation. These molecules include fibrin, fibronectin, and vitronectin [3].

A solution of PRP is prepared by first harvesting the patient’s own blood, often from the median cubital vein [4]. This autologous blood is then centrifuged to allow for the separation of the various components based on the relative density. This allows for separation and collection of the platelet-poor plasma from the other components of blood [4]. Further centrifugation allows for the separation and collection of the buffy coat layer that contains PRP and/or leukocytes [4]. The prepared PRP is then re-administered to the site of injury.

### What are the different formulations and types of platelet-rich plasma?

PRP either be activated or non-activated and be either leukocyte-rich or leukocyte-poor. Based on these four variations, a classification system of PRP has been proposed (Table 1) [7]. To be considered activated, PRP is prepared with calcium chloride with or without thrombin [5, 7, 8]. This activation leads to the release of cytokines from the granules in platelets and thus, ensures that they will be in abundance upon injection of the preparation [8]. On the other hand, the non-activated form depends on platelet contact with intrinsic collagen and thromboplastin, which then activate the platelets within connective tissue [8]. Whether activated prior to injection or non-activated, it is important to note that 70% of the growth factors are secreted within ten minutes of activation and 100% of growth factors are secreted within one hour of activation [8].

**Table 1.** Types of platelet-rich plasma.

	White blood cells	Activation	Platelet concentration	
Type 1	Increased	None	A, >5×	B, <5×
Type 2*	Increased	Activated	A, >5×	B, <5×
Type 3**	Minimal or None	None	A, >5×	B, <5×
Type 4**	Minimal or None	Activated	A, >5×	B, <5×

Table adapted from Mishra et al. (2012) [7].

* Type 2 is also referred to as platelet leukocyte gel;

** Type 3 is also referred to as platelet concentrate;

*** Type 4 is also referred to as platelet gel;

A, above; B, below.

As stated above, PRP can also be leukocyte-rich or leukocyte-poor. Although the role of leukocytes within PRP is not fully understood, it has been thought that the leukocytes play a role in inhibiting some bacterial growth and improving soft tissue healing which has been complicated by infection [8]. On the other hand, the leukocytes have also been proposed to cause an exaggerated inflammatory response as they stimulate the release of interleukin (IL)-1β, IL-6, IL-8, and tumor necrosis factor alpha (TNF-α) [8]. Furthermore, leukocytes have also been thought to stimulate the production of reactive oxygen species that can lead to further muscle damage and inflammation [8].

### What does the basic science evidence suggest?

On a molecular level, for degenerative conditions such as osteoarthritis, *in vitro* studies have found that the use of PRP stimulates chondrocytes and synoviocytes to produce the cartilage matrix while also downregulating key molecules that are mediators of the inflammatory response, such as IL-1 [9, 10]. Additionally, studies have shown that PRP also increases proteoglycan and type II collagen synthesis, two biological molecules which are important for structural organization of the cartilage framework, through increased mRNA gene expression [10, 11]. This anabolic effect of PRP has been thought to be closely linked to promoting TGF-β production, a catabolic cytokine, which decreases type I collagen gene expression. Further downstream, this leads to an increase in type II collagen and aggrecan gene expression [12]. Furthermore, TGF-β has been found to be needed throughout the entire process of differentiation of bone marrow stromal cells to cartilage [13]. A systematic review of the basic science evidence on the use of PRP for osteoarthritis was conducted in 2013 by Smyth et al. [11]. In their review, 15 of the 21 *in vivo* and *in vitro* studies included reported that PRP promotes the proliferation of chondrocytes, increases proteoglycan production, and leads to a greater deposition of type II collagen. To compound this effect, PRP was also found by three of the studies to promote chondrocyte viability and by five of the studies to promote mesenchymal stem cell differentiation.

PRP has also been found to have similar effects on damaged tendons to promote accelerated repair. Tendons are known to have low metabolic rates and thus, tend to heal slowly after injury. Studies on severed sheep tendons have shown that PRP promotes the proliferation and secretion of VEGF and hepatocyte growth factor, both of which stimulate angiogenesis and reduce inflammatory fibrosis [14, 15]. Similar has also been observed in rat models as PRP has been found to promote both tendon-to-bone healing and remodeling [16]. Furthermore, in an extensive study on human rotator cuff tendons by Jo et al. (2012) [17], it was found that PRP activated with calcium significantly increased the gene expressions of decorin and tenascin-C, a proteoglycan and glycoprotein of tendon, respectively. In addition, the study reported that PRP led to an increased synthesis of collagen and glycosaminoglycan [17].

In contrast to the above, the *in vivo* and *in vitro* evidence for ligamentous injury appears to be conflicting. For instance, Dhillon et al. (2015) harvested 11 human anterior cruciate ligament (ACL) remnants and evaluated the effect of different concentrations of PRP and platelet-poor plasma (PPP) on their cell growth [18]. The authors observed that the addition of PRP or PPP did not increase cell viability in many of the samples but rather, it increased the rate of apoptosis in comparison to control. Furthermore, larger concentrations of PRP and PPP were found to have more pronounced effects and higher rates of apoptosis. However, there are also studies which suggest the contrary. In animal models, PRP has been found to enhance the growth of ligamentous cells [19–22]. Similar has also been found in human harvested grafts where the addition of PRP has been linked to increased concentrations of TGF-β, PDGF, EGF, VEGF, and an overall increase in the gene expression of type III collagen [23].

### What does the clinical evidence suggest?

#### Knee osteoarthritis

Osteoarthritis of the knee is one of the most common disorders in the elderly, with approximately 10–15% of individuals aged 60 years and above suffering from the condition [10, 24, 25]. Without adequate treatment, the condition progresses continuously due to cartilage damage and inflammatory changes [25]. This gradual progression is due to the limited regeneration potential of articular cartilage. Thus, repetitive trauma, injuries, and aging lead to thinning of the joint space and eventually, limited and painful joint movement [10]. The treatment of knee osteoarthritis centers around both surgical and non-surgical options; however, non-surgical treatment has recently garnered greater attention. There are several different options which have been extensively studied that have been shown to be effective at symptom relief: these include non-steroidal anti-inflammatory drugs (NSAIDs), intra-articular injections, corticosteroids, and more recently, PRP [25].

Due to the basic science evidence suggesting that the use of PRP can have beneficial effects on a molecular level, there has been a profound growth in the number of randomized trials and meta-analyses that evaluate whether this promise is translated to a clinical level. Current guidelines from the American Academy of Orthopaedic Surgeons (AAOS) and National Institute for Health and Care Excellence (NICE) suggest that the evidence is inconclusive in regard to the use of PRP for knee osteoarthritis; however, several meta-analyses have been conducted on the topic and they provide important results for the practitioner. Laudy et al. (2015) conducted a systematic review and meta-analysis of ten studies, which included five randomized controlled trials (RCTs) and five non-randomized trials [26]. Although statistical pooling was limited due to the wide variation in outcome reporting, the authors found that PRP injections led to a reduction in pain scores and an improvement in function at six-month follow-up. Adding to the results of this review, Riboh et al. (2016) conducted a network meta-analysis of six RCTs and three prospective comparative trials, totaling 1055 patients [27]. Here they found that both leukocyte-rich and leukocyte-poor PRP led to reductions in mean Western Ontario and McMaster Universities Arthritis Index (WOMAC) scores at final follow-up time; however, only leukocyte-poor PRP displayed significant reductions in comparison to both placebo and hyaluronic acid. Even though both meta-analyses found benefits in favor of PRP, they suffered from evidence which had a considerable risk of bias and variability in reporting.

More recently, two meta-analyses conducted by Dai et al. (2016) [28] and Shen et al. (2017) [25] reported results which provided a clearer picture as to the benefits of PRP for knee osteoarthritis. In their analysis of 10 RCTs, Dai et al. (2016) [28] found that PRP led to significant improvements in WOMAC VAS pain scores and functional scores at 12-month follow-up by 2.83 points and 12.53 points, respectively, in comparison to hyaluronic acid alone. Importantly, both estimates surpassed the minimal clinically important difference threshold. Yet, significant effects were not found at shorter follow-up times, most notably six months [25, 28]. The results of this review should be contrasted with that of Shen et al. (2017) [25] who included an additional four RCTs, for a total of 1423 patients. In their analysis, it was found that PRP led to significant improvements in WOMAC scores at shorter follow-up times as well, namely at three- and six-month follow-up. It is important to note, however, that both meta-analyses reported significant heterogeneity in their outcomes.

#### Rotator cuff repair

Rotator cuff tears are the most common cause of shoulder disability with their prevalence ranging from 20 to 40% in the aging population [29, 30]. Surgical and non-operative options exist for the treatment of rotator cuff tears; however, the optimal strategy is largely dependent on the grade of the tear. Both options have been found to have varying levels of success with close to 90% of arthroscopic repairs having good to excellent results at 10-year follow-up and non-operative treatment having approximately 50–60% satisfactory results [31–33]. With the prevalence of these injuries rising and an increased emphasis placed on finding optimal treatment strategies to prevent re-tear while continuously improving patient outcomes, there has been a large body of evidence focused using PRP supplementation to potentially promote rotator cuff healing.

Current guidelines have not yet assessed whether PRP supplementation should be used by clinicians for existing treatment strategies. Even though basic science evidence has largely found that that PRP has beneficial effects for tendinous injuries, clinical trials and meta-analyses have shown inconsistent results. A recent Cochrane review of 19 randomized trials assessed the use of PRP supplementation for various orthopedic clinical conditions, one of which included rotator cuff repair [34]. In their analysis, six studies were included which analyzed the use of PRP for small, moderate, and complete rotator cuff tears. One year after surgery, the authors found that there was no significant difference in Constant-Murley and L’Insalata functional scores when PRP was added to surgical repair; this analysis, however, did come with significant heterogeneity [34]. Similar was also seen with pain as PRP only led to a 0.69-point decrease and a 0.29-point decrease in VAS scores at three-month and one-year follow-up, respectively, in comparison to control [34]. Interestingly, the risk of re-tear at one-year follow-up was 45% less in patients who received PRP supplementation, however, this was also found to be non-significant [34].

Adding to the results of the above review, a meta-analysis conducted by Vavken et al. (2015) included an additional seven trials [35]. Overall, only a 13% decrease in the risk of re-tear rate was found when PRP was used [35]. Upon subgroup analysis, it was found that tears ≤3 cm had a significantly reduced risk of re-tear by 40% when PRP was used [35]. This difference was not observed for larger tears >3 cm [35]. Although functional and pain outcomes were not assessed by this meta-analysis, several additional meta-analyses were conducted which found similar results to the Cochrane review [36, 37].

Recently, Saltzman et al. (2016) conducted a review of seven conflicting meta-analyses on the topic [38]. All the included meta-analyses in their review varied in regard to the clinical outcomes assessed and follow-up times measured; however, the quality of each remained consistent as all had scores >15 on the Quality of Reporting of Meta-analyses (QUOROM) checklist [38]. Six of the seven meta-analyses reported no significant difference in Constant-Murley and American Shoulder and Elbow Society (ASES) functional scores [38]. Similarly, no difference was found in VAS pain scores in five of the seven studies included and in overall re-tear rates, regardless of the tear size [38].

#### Epicondylitis

Epicondylitis can be divided into two broad categories, namely lateral epicondylitis (tennis elbow) and medial epicondylitis (golfer’s elbow). The prevalence of each of these conditions has been found to range from 2 to 3% in the general population and even higher in those between 40 and 60 years of age [39, 40]. Both conservative and interventional treatment options exist for epicondylitis. Like knee osteoarthritis, patients may receive NSAIDs, corticosteroids, physical therapy, or even extracorporeal shock wave therapy [41]. Surgery is also an option for patients who have persistent symptoms despite continued efforts at conservative treatment [41]. Recently, PRP has garnered greater attention as a potential long-term treatment option for lateral epicondylitis.

Currently, there is little high-quality evidence on the use of PRP for medial epicondylitis; however, there have been several meta-analyses conducted on the topic for lateral epicondylitis. In the Cochrane review of conducted by Moraes et al. (2014), three trials were included that assessed the use of PRP supplementation in comparison to either autologous whole blood or dry needling for lateral epicondylitis [34]. It was found that PRP provided no significant functional improvement at three-month follow-up in comparison to control. Due to the limited number of studies published on the topic at the time of their review, further outcome analysis was limited.

A more recent network meta-analysis conducted in 2016 by Arirachakaran et al. included 10 RCTs, of which seven compared PRP to either autologous blood or steroids [42]. It was found that PRP led to a significant reduction in VAS pain scores by 0.54-point comparison to steroids [42]. This significant effect was even more pronounced at the latest measured follow-time as there was a 2.02-point reduction [42]. On the other hand, when PRP was compared with autologous blood, significant differences were not observed at three-month and latest measured follow-up times [42]. Functional outcomes, as measured by the Disabilities of the Arm, Shoulder, and Hand (DASH) score, also displayed a significant benefit in favor of PRP in comparison to both steroids and autologous blood at three-month follow-up; however, this effect was only maintained at the latest follow-up time when PRP was compared with steroids [42].

#### Patellar tendinopathy

Patellar tendinopathy, or commonly known as “Jumpers Knee,” is a frequent cause of anterior knee pain among athletes, accounting for approximately 14.2% of all sports-related injuries [43]. As the name implies, the condition is due to repetitive jumping movements and other similar movements that cause increased tendinous loading. Similar to epicondylitis, several non-conservative treatment options have been evaluated, including quadriceps strengthening exercises, low-intensity shockwave therapy, and corticosteroids [44, 45]. For cases that are chronic and refractory to treatment, arthroscopic repair is also used [43]. PRP, due to its potential to promote repair and reduce inflammation, has also been recently investigated as a potential conservative treatment strategy.

Currently, there is limited high-quality evidence on the topic; however, a meta-analysis conducted in 2015 by Liddle and Rodríguez-Merchán included a total of 11 studies, two of which were RCTs [46]. Statistical pooling of these RCTs did not reveal any significant difference in regard to Victoria Institute of Sport Assessment (VISA) questionnaire and VAS pain scores at six-month follow-up [46]. The strength of these conclusions was limited due to the lack of high-quality evidence on the topic.

#### Achilles tendinopathy

Achilles tendinopathy is a common overuse sports injury and is often associated with severe pain and swelling at the tendinous insertion site [47]. The incidence of the condition has been cited to be close to 25% among athletes worldwide [48]. Furthermore, without adequate treatment, the situation can get worse as tendon rupture has been reported to occur in 8% of cases [48]. As for patellar tendinopathy, the treatment revolves around muscle strengthening exercises and anti-inflammatory medications; however, due to the limited repair potential of tendons, PRP has also been proposed as a treatment option.

Two recent systematic reviews were published in 2015 on the topic of treatment strategies for Achilles tendinopathy by Maffulli et al. [49] and Di Matteo et al. [47]. Each of these reviews contained two RCTs and one RCT, respectively, on the topic. In their qualitative synthesis of the results from these trials, both reviews found that PRP did not lead to significant improvements in VISA scores or ultrasonographic score at one-year follow-up in comparison to control [47, 49]. Again, similar to patellar tendinopathy, the strength of these conclusions was limited due to the lack of high-quality evidence on the topic.

#### Hamstring injuries

Acute hamstring injuries have been cited to account for approximately 29% of all sports-related injuries [50, 51]. With this type of injury being one of the most common in the sports realm, there has been a great focus on optimizing treatment strategy to improve return to play time. Broadly, there are two types of acute hamstring injuries. Type 1 strains commonly occur during sprinting when the hamstring muscles quickly contract after lengthening, whereas Type 2 strains occur during prolonged and excessive lengthening of the hamstring muscles [51]. With conservative treatment, the injury usually resolves within three to six weeks; however, recently PRP injection has been studied to potentially quicken recovery times.

To date, there has been one meta-analysis conducted by Pas et al. (2015) which evaluated PRP injections in addition to other conservative approaches for hamstring injuries [52]. In their meta-analysis of 10 RCTs, they included three trials which compared the use of PRP injection and physiotherapy with either no injection, PPP, or placebo [52]. It was found that PRP injection with physiotherapy only led to a 3% greater return to play rate in comparison to control; this difference did not reach significance [52]. The authors noted that this analysis also came with a significant level of heterogeneity [52]. Although statistical pooling was not performed for pain, the authors noted in their qualitative review that studies were not reporting a significant difference in change of pain scores comparison to control.

#### Anterior cruciate ligament repair

It is estimated that close to 75,000 anterior cruciate ligament (ACL) repairs occur in the United States yearly [53]. With advances in surgical approaches and graft selection, the outcomes after surgery are becoming much better; however, the failure rate has still been reported to be close to 14% [54]. To further enhance the repair of ligamentous injuries, PRP has been used as a part of minimally invasive options to promote ACL healing and earlier return to work.

In the Cochrane review conducted by Moraes et al. in 2014, there were four RCTs included which assessed the use of PRP in ACL repair [34]. Although statistical pooling was not performed for this group of patients due to the high variability in outcome reporting, qualitatively it was found that all four studies did not report a significant difference versus control in regard to International Knee Documentation Committee (IKDC) functional results at one-year follow-up [34]. Adding to the results of this review, Figueroa et al. (2015) conducted a systematic review of 11 studies, of which nine were RCTs that evaluated the use of PRP in ACL surgery [55]. Again, although statistical pooling was not performed, the authors noted that five of the six studies measuring clinical outcomes did not report a significant difference with the addition of PRP [55]. Similarly, five of the six studies reported no significant difference in tunnel healing or widening with the addition of PRP [55].

## Key evidence based themes for the clinician

**Figure 1.**
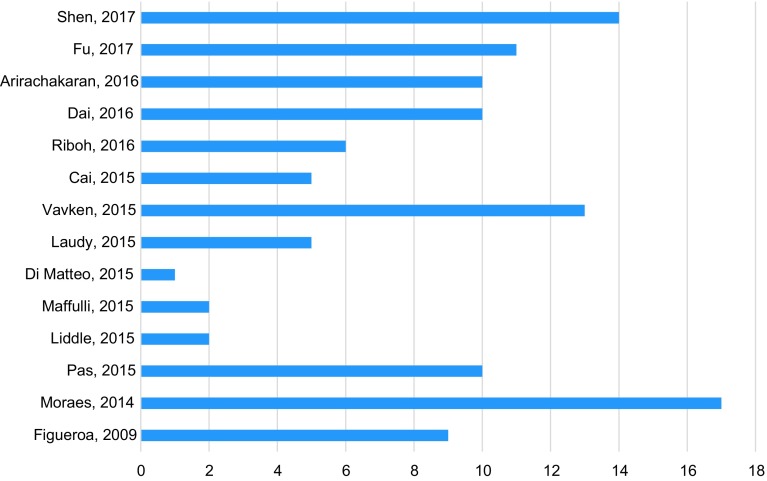
Number of level-I studies related to PRP in included meta-analyses.

For the clinician wishing to utilize PRP in practice, there are several important considerations that need to be made based on the conflicting meta-analyses for different conditions and the paucity of guidelines developed by professional organizations. Based on our review, it appears that PRP may provide some benefit in patients who have knee osteoarthritis and lateral epicondylitis. On the other hand, the evidence appears to be inconsistent or displaying a minimal benefit for PRP usage in rotator cuff repair, patellar and Achilles tendinopathies, hamstring injuries, ACL repair, and medial epicondylitis. With this information in hand, it is imperative to note that thus far, several of the meta-analyses that have been conducted to date have had limited statistical pooling and subgroup analyses. Future meta-analyses should focus on performing subgroup analysis based on the type of PRP preparation used to determine whether the effects differ based on the different formulations. Furthermore, many of the RCTs included in these analyses have been found to have a high risk of bias which limits external validity. A clinician wishing to utilize evidence-based practice may want to consider using PRP in their patient population; however, they should be weary that the evidence appears to be inconsistent and thus far, inconclusive (Figure 1).

## Conflict of interest

The authors declare that they have no conflict of interest.
